# Depression in a 15-Year-Old With Metastatic Ewing Sarcoma: A Case Report

**DOI:** 10.1192/j.eurpsy.2026.11552

**Published:** 2026-07-31

**Authors:** S. Khalki

**Affiliations:** Pediatrics, Farhat Hached Hospital, Sousse, Tunisia

## Abstract

**Introduction:**

Ewing sarcoma is a rare and malignant bone tumor, affecting especially adolescents and children. It has a high metastatic potential, heavily influencing its prognosis. Beyond the physical burden of the disease, the psychological impact can also be as profound.

This case report tackles the emotional and psychiatric impact of a recently diagnosed metastatic Ewing Sarcoma in a 15 year old patient. Shortly after disclosure of the diagnosis, the patient began exhibiting depressive symptoms such as sadness, crying, insomnia, social withdrawal, non engagement in his treatment, and loss of interest in previously enjoyed activities.

**Objectives:**

This case report aims to:

Illustrate how depression can follow advanced pediatric cancer, specifically metastatic Ewing sarcoma Identify early depression manifestations in such patients Highlight the importance of early on psychological care

**Methods:**

To assess the patient’s psychological state, we used two validated scales. The Hospital Anxiety and Depression Scale (HADS) using the approved Arabic version, was selected as a self report tool suitable for hospitalized patients enabling the screening for both anxiety and depressive symptoms.

Furthermore, we used the Hamilton Depression Rating Scale (HAM-D) to assess the severity of the symptoms presented.

**Results:**

Our patient scored 5 on the HADS anxiety subscale, falling within the normal range, whereas he scored 11 on the HADS depression subscale, indicating clinically significant depressive symptoms.The most relevant symptoms were his inability to enjoy the things he used to enjoy, and his difficulty to feel cheerfulness.

Further assessment with the HAM-D score indicated a result of 21, which corresponds to a moderate depressive episode, with the highest noted symptoms being his inability to perform routine chores unassisted, weight loss and insomnia.

These findings are compatible with existent literature. In fact, a meta analysis by Wu et al. found that the prevalence of depression among children and adolescents with cancer is approximately 20.4%. Besides,in a prospective study of pediatric oncology patients in Oman,by Al‑Saadi LS,Chan MF, 56.5% showed depressive symptoms within three months of diagnosis.

The moderate depression evaluated in our patient adds a clinical relevance to these findings. It endorses the fact that depressive symptoms in adolescents facing aggressive cancers, like metastatic Ewing sarcoma, are not rare, on the contrary, they should be expected and assessed preliminarily for a global approach in patient care.

**Conclusions:**

When facing life-threatening illnesses like metastatic Ewing sarcoma, clinical focus tends to center on somatic care, often overlooking the psychological impact.

This case report highlights the intertwined liaison between physical illness and mental health in pediatric oncology; and emphasizes the urgent need for early detection of depressive symptoms and the importance of early on psychological care.

**Disclosure of Interest:**

None Declared

